# Sensory reweighting and self-motion perception for postural control under single-sensory and multisensory perturbations in older Tai Chi practitioners

**DOI:** 10.3389/fnhum.2024.1482752

**Published:** 2024-11-01

**Authors:** Xiao-xia Liu, Guozheng Wang, Ruixuan Zhang, Zhuying Ren, Di Wang, Jun Liu, Jian Wang, Ying Gao

**Affiliations:** ^1^Department of Sports Science, College of Education, Zhejiang University, Hangzhou, China; ^2^Taizhou Key Laboratory of Medical Devices and Advanced Materials, Taizhou Institute of Zhejiang University, Taizhou, China; ^3^Key Laboratory for Biomedical Engineering of Ministry of Education, College of Biomedical Engineering and Instrument Science, Zhejiang University, Hangzhou, China; ^4^Center for Psychological Science, Zhejiang University, Hangzhou, China

**Keywords:** multicomponent mind–body exercise, multiscale entropy, postural balance, perceptual illusion, sensory reweighting, wavelet transform

## Abstract

**Purpose:**

Impairment in perception and sensory reweighting could predispose older adults to falls. This exploratory study aimed to investigate the differences in sensory reweighting and self-motion perception for postural control under single-sensory and multisensory perturbations between older Tai Chi (TC) practitioners and healthy active older controls.

**Methods:**

Twenty-four TC practitioners and 23 age-matched non-practitioners were recruited in this observational study. Participants stood on a force plate with or without a foam surface (baseline), followed by 36 s of visual rotation stimuli, vestibular rotation stimuli, or reduced somatosensory input (adaptation), and then continued standing for 44 s (reintegration). The center-of-pressure (COP) trajectories and self-motion perception were recorded. COP signals were analyzed using traditional sway, multiscale entropy, and wavelet analysis methods and the time-window-of-integration model to examine the postural balance performance and the flexibility and speed of sensory reweighting.

**Results:**

Significant interaction effects of group with sensory perturbation and group with time window on COP parameters were observed (*p* < 0.05). Compared with non-practitioners, TC practitioners exhibited higher postural stability and complexity as the difficulty of standing tasks increased and smaller time windows to return to baseline levels as disturbance time evolved. Moreover, TC practitioners exhibited significantly greater weighting on unperturbed sensory systems, lower weighting on perturbed sensory systems for postural control, and higher self-motion perception ability under visual, vestibular, and visual-vestibular perturbations (*p* < 0.05).

**Conclusion:**

Long-term TC practitioners exhibited superior postural stability and adaptability under challenging sensory perturbations, and smaller amplitudes and shorter durations of postural aftereffects over time during adaptation and reintegration. These improvements may be partly attributed to more rapid and flexible sensory reweighting and improved self-motion perception for postural control.

## 1 Introduction

Falls and related injuries in older adults are becoming increasingly common worldwide, making their prevention and management a critical global challenge (Montero-Odasso et al., 2022; Seppala and van der Velde, 2022; World Health Organization, 2021). Balance impairment is a strong risk factor for falls among older adults (Ambrose et al., 2013; Song et al., 2021) and becomes more prominent with aging (Dijkstra et al., 2020; Völter et al., 2021), imposing a high socioeconomic burden on aging societies (Dijkstra et al., 2020). Balance control is a complex sensorimotor process that requires the nervous system to successfully integrate multisensory information (visual, vestibular, and somatosensory) to accurately perceive the current postural state (Craig and Doumas, 2019; Horak, 2006; Ma et al., 2022). Multisensory integration is achieved through a process known as sensory reweighting, in which the brain determines the importance (weighting) of a sensory channel based on its relative reliability in the current context (Craig and Doumas, 2019; Ernst and Banks, 2002; Peterka and Loughlin, 2004). For example, when the reliability of one or more senses diminishes, the brain must rely more on the remaining intact sensory systems to achieve stable balance (Ma et al., 2022; Wang et al., 2022). The flexible and adaptive reweighting of sensory information is crucial for postural stability (Ketterer et al., 2022). However, sensory reweighting as a specific mechanism is highly sensitive to age-related decline (Craig and Doumas, 2019; Doumas and Krampe, 2010). Older adults usually experience larger amplitudes and longer durations of postural instability during sensory transitions than those of younger adults owing to inefficient and inflexible sensory reweighting (Chancel et al., 2018; Doumas and Krampe, 2010; Zhang et al., 2020). Additionally, older adults are more susceptible to irrelevant visual information due to excessive visual dependence, leading them to more frequently experience multisensory perceptual illusions compared to younger participants (Craig and Doumas, 2019; Jones and Noppeney, 2021). Balance exercises that specifically target multisensory integration mechanisms may lead to significant improvements in sensory reweighting and perception for postural control (Allison et al., 2018; Bugnariu and Fung, 2007; Ketterer et al., 2022).

Tai Chi (TC), a form of balance training, offers unique advantages in enhancing the plasticity of the sensorimotor system and benefiting the nervous system (Chen et al., 2021; Shao et al., 2022; Zhang et al., 2021). Several studies have reported the clinical utility of TC for counteracting balance dysfunction caused by various diseases and conditions, such as sarcopenia (Huang et al., 2022), frailty (Courel-Ibáñez et al., 2022), Parkinson’s disease (Li et al., 2022), osteoarthritis (Wang et al., 2016), chronic diseases (Wang et al., 2022; Yeh et al., 2020), and cognitive and movement impairments (Chen et al., 2023; Lei et al., 2022). To date, TC has been recommended by multiple global organizations as a cost-effective and safe exercise therapy for fall prevention and management in older adults (Panel on Prevention of Falls in Older Persons, American Geriatrics Society and British Geriatrics Society, 2011; Centers for Disease Control and Prevention, 2023; Montero-Odasso et al., 2022; World Health Organization, 2021). Nevertheless, the long-term effects of TC on postural control and some of the related mechanisms, especially those related to sensory reweighting, remain unknown.

Sensory reweighting and self-motion perception for postural control can be measured using sensory manipulations to challenge the nervous system (Chancel et al., 2022; Ketterer et al., 2022). This involves creating sensory conditions that are more challenging by further modifying the visual, vestibular, and/or somatosensory inputs (Ketterer et al., 2022; Doumas and Krampe, 2010). During sensory perturbations, the nervous system must change its reliance, or “weighting,” on different sensory inputs to perceive orientation and maintain stability (Doumas and Krampe, 2010; Ma et al., 2022). Previous studies have indicated that older adults with at least one year of TC experience showed improved postural stability under more challenging sensory conditions (Tsang et al., 2004; Tsang and Hui-Chan, 2006). Two recent cross-sectional studies reported that older adults with long-term TC practice (at least five years) exhibited better sensory reweighting when standing on a firm surface with their eyes closed (Cui et al., 2024) and demonstrated higher perceptual abilities under the fusion illusion condition (Wang et al., 2023). However, one limitation of previous TC-related studies is that they primarily employed long-duration, constant, or static sensory perturbations, such as eyes open/closed, single-leg stance, or tandem stance tasks, which allowed participants to reach steady-state conditions (Cui et al., 2024; Wayne et al., 2014). These traditional perturbation paradigms involve less dynamic sensory reweighting and multisensory illusion (Doumas and Krampe, 2010; Ketterer et al., 2022). In fact, falls in older adults are often caused by sudden sensory changes in dynamically changing environments. Recently, virtual reality (VR), rotating platforms, and variable support surfaces have provided novel opportunities for researchers to systematically modulate visual, vestibular, and somatosensory inputs in an arbitrary yet standardized manner (Chander et al., 2019; Hedges et al., 2011; Li et al., 2019; Wang et al., 2022; Wang G. et al., 2024). These technologies can be used to induce more challenging balancing tasks that impose additional demands on sensory reweighting and balance control (Ketterer et al., 2022; Martelli et al., 2019), which are necessary for revealing the underlying mechanisms of balance control (Doumas and Krampe, 2010).

Another limitation of most previous TC-related studies is the assessment of postural control using only the traditional sway parameter of the center-of-pressure (COP) derived from single-timescale techniques (Wayne et al., 2013, 2014). However, standing postural control is regulated by multiple neurophysiologic processes that include the functional integration of multiple sensory inputs, spinal and supraspinal neural networks, the peripheral neuromuscular system, and cognitive functions (Zhou et al., 2023). Postural control contains meaningful information across multiple timescales, which can be captured by metrics derived from complexity theory, but not by traditional single-scale metrics (Busa and van Emmerik, 2016; Zhou et al., 2023). Therefore, multiscale entropy (MSE) techniques based on the principles of information theory have been introduced to the study of postural control to quantify how individuals regulate their postural fluctuations at multiple time scales (Busa and van Emmerik, 2016). The rationale for the use of MSE is that healthy systems display dynamics indicative of a highly adaptable network of neuromuscular connections (Busa and van Emmerik, 2016; Li et al., 2023; Zhou et al., 2023). MSE measures enable the estimation of the amount of “complexity” in a postural control system, whereby an increase in MSE values indicates a system exhibiting a greater degree of complex dynamics (Busa and van Emmerik, 2016; Lipsitz and Goldberger, 1992). Wayne et al. (2014) reported that MSE offers a complementary approach to traditional COP measures for characterizing COP complexity when standing and may be more sensitive to the effects of TC in healthy adults. However, the effects of TC on the COP complexity of standing postural control under single-sensory and multisensory perturbation conditions in older adults are relatively unclear.

Furthermore, time-domain measures of the COP, such as mean distance and velocity, reflect only the final integration of the sensorimotor systems, without indicating the underlying mechanism (Lin et al., 2019). Balance performance may not be reflected by time-domain measures when people use various control strategies (Carpenter et al., 2001; Lin et al., 2019). Therefore, in previous studies, the time-frequency approach was commonly used to investigate the effect of age on the balance mechanism (Richer and Lajoie, 2020). The wavelet transform, a nonlinear time-frequency analysis of postural control, decomposes COP signals into different frequency bands, indicating the contribution of each sensory system to posture modulation, thus providing a better understanding of sensorimotor integration in postural control (Cui et al., 2024; Lafleur and Lajoie, 2023; Lin et al., 2019). Specifically, the energy content of the COP signal can divided into four distinct frequency bands, namely, the moderate-frequency (1.56–6.25 Hz), low-frequency (0.39–1.56 Hz), very-low-frequency (0.10–0.39 Hz), and ultralow frequency (<0.10 Hz) bands, which represent the muscular proprioception, cerebellar, vestibular, and visual systems, respectively (Cui et al., 2024; Lafleur and Lajoie, 2023; St-Amant et al., 2020). To date, only a few studies have applied this time-frequency analysis to investigate changes in the postural control mechanisms of older adults with long-term TC practice.

The aim of this exploratory study was to conduct cross-sectional comparisons between long-term TC practitioners and healthy active older adults using traditional linear and novel nonlinear methods to investigate the differences between the groups in sensory reweighting and self-motion perception for postural control under single-sensory and multisensory perturbations. We hypothesized that long-term TC practitioners would exhibit superior postural responses and self-motion perception and greater effectiveness in sensory reweighting for postural control than non-practitioners.

## 2 Materials and methods

### 2.1 Participants

Forty-eight older adults (aged 60–83 years) were recruited by oral presentations, flyers, and word-of-mouth at multiple community sports centers. The inclusion criteria were as follows: (1) age ≥ 60 years; (2) independently ambulatory without the use of assistive devices; and (3) long-term TC experience (at least 3 days per week, ≥ 30 min each session, for more than 6 years) for the TC group and no previous TC experience, but general aerobic exercise experience such as walking (at least 3 days per week, ≥ 30 min each session, for more than 6 years) for the control group. The exclusion criteria for both groups were as follows: (1) a history of sensory, neurological, or musculoskeletal injury affecting balance ability; (2) cardiovascular pathologies, motion sickness, dizziness, vertigo, or any other vestibular disorders; (3) self-reported psychological problems related to fall risks, such as fear of falling, anxiety, or depression; (4) Montreal Cognitive Assessment (MoCA; Bernstein et al., 2011) score < 25; and (5) Berg Balance Scale (BBS, Bogle Thorbahn and Newton, 1996) score < 45. All participants were right-hand dominant (defined by the hand they stretched out to reach an object; Haddad et al., 2008), right-leg dominant (defined using the kickball test; Lu et al., 2013), and had normal or corrected vision. The purpose and procedures of this study were explained at the beginning of the study, and written informed consent was obtained from all participants before participation. Each participant received financial compensation for their time. This study was approved by the Institutional Review Board and adhered to the ethical standards of the Declaration of Helsinki.

### 2.2 Experimental design

**Figure 1:**
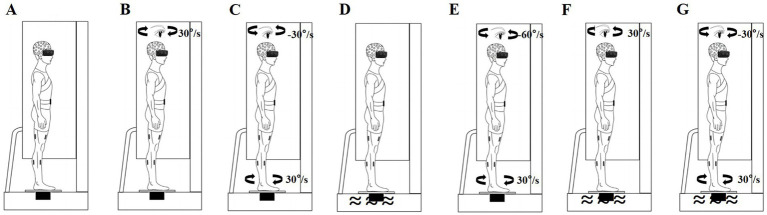
Schematic of sensory perturbations. **(A)** Baseline non-perturbation; **(B)** Visual perturbation; **(C)** Vestibular perturbation; **(D)** Somatosensory perturbation; **(E)** Visual-vestibular perturbation; **(F)** Visual-somatosensory perturbation; and **(G)** Vestibular-somatosensory perturbation.

We used a VR helmet (VIVE PRO 2; HTC Corp., Taoyuan City, Taiwan), rotating platform (All Controller; Nanjing, China), and foam surface (Airex AG; Sins, Switzerland) to perturb the participants’ sensory systems. Rotating visual scenes (Chander et al., 2019; Ketterer et al., 2022; Wang G. et al., 2024), head-in-space acceleration induced by platform rotations (St George and Fitzpatrick, 2011; Yang et al., 2023), and soft surfaces (Bai et al., 2023; Li et al., 2019) reportedly can effectively disrupt visual, vestibular, and somatosensory inputs, respectively. All participants performed standing balance tasks under baseline non-perturbation and six sensory perturbations, with each task lasting 100 s and a 5-min rest period between conditions. To avoid training effects, each participant completed only one trial for each standing balance task (Maitre et al., 2015; Wang et al., 2022; Wang G. et al., 2024). After baseline non-perturbation testing, standing balance was measured under the following six randomized sensory perturbation conditions (Maitre et al., 2015; Wang et al., 2022; Wang G. et al., 2024). (1) Baseline condition: the participants wore the VR helmet and performed standing tasks without perturbations (Figure 1A). (2) Visual perturbation: after 20 s of non-perturbation, the VR visual scene began to move and stopped after 36 s of clockwise uniform rotations at 30°/s; thereafter, the participants continued non-perturbed standing for 44 s (Figure 1B). (3) Vestibular perturbation: after 20 s of non-perturbation, the platform began to move and stopped after 36 s of clockwise uniform rotations at 30°/s; subsequently, the participants continued non-perturbed standing for 44 s (Figure 1C). (4) Somatosensory perturbation: the foam surface was used for 100 s (Figure 1D). (5) Visual-vestibular perturbation: after 20 s of non-perturbation, the VR visual scene was displayed and rotated counterclockwise at 60°/s while the platform rotated clockwise at a speed of 30°/s for 36 s; the participants then continued non-perturbed standing for 44 s (Figure 1E). (6) Visual-somatosensory perturbation involved a combination of the visual and somatosensory perturbations listed in points 2 and 4 (Figure 1F). (7) Vestibular-somatosensory perturbation involved a combination of the vestibular and somatosensory perturbations listed in points 3 and 4 (Figure 1G).

Acceleration and deceleration of the platform and visual scene were completed within 2 s. Prior to formal testing, a brief trial was performed to familiarize the participants with the experimental protocol (Maitre et al., 2015; Wang et al., 2023). The participants were instructed to keep their eyes open (allowing normal blinking) and look straight ahead under all conditions, which was ensured using an eye-tracking system built into the VR headset. They were asked to stand barefoot on a force plate with their feet shoulder-width apart, maintain a normal posture, and remain as still as possible in each condition. A force plate, with or without a foam surface, was placed at the center of the rotating platform. In the first trial, the foot position was marked with a tape to ensure consistent placement throughout the study. During all postural tasks, the participants were secured with a safety harness to prevent falls or injuries. Two investigators stayed close to the participants throughout the test to prevent them from falling. Once the participants moved their feet or fell, the trial was stopped, and the results were excluded from further analyses (Hao et al., 2021). Symptoms of discomfort, such as general discomfort, fatigue, eyestrain, and vertigo, were evaluated immediately after each task using the VR sickness questionnaire, which has high reliability (Kennedy et al., 1993; Kim et al., 2018). If a participant reported any severe discomfort, the trial was stopped.

### 2.3 Data collection and preprocessing

A Wii balance board (Nintendo, Kyoto, Japan) was used to record the COP with a sampling frequency of 100 Hz. Accumulating evidence has demonstrated excellent reliability and validity of COP measurements (Huang et al., 2022; Leach et al., 2014). To investigate the differences in self-motion perception between older TC practitioners and non-practitioners, a mid-experiment survey was conducted to assess the participants’ perception of body rotation (Wang G. et al., 2024). After completing each standing task, the participants were asked the following questions: (1) Did you perceive your body as rotating? Please select Yes or No. (2) In what direction did your body rotate? Please select the clockwise or counterclockwise direction. When both responses were consistent with the actual situation, their self-motion perception was considered correct.

COP data were processed using MATLAB R2021b software (MathWorks, Natick, MA, United States). Data from the acceleration or deceleration phases of the rotation were discarded, and the remaining data were filtered using a 20 Hz low-pass, second-order, zero-lag Butterworth filter (Wang et al., 2022). The mean of the filtered data was subsequently removed (Wang et al., 2022). COP data were analyzed using traditional linear (COP sway: velocity and root mean square [RMS]) and nonlinear (MSE, discrete wavelet transform) methods. Sway velocity is considered a reliable dynamic indicator of the efficiency of posture control and was calculated by dividing the COP excursion distance by the duration time (Hao et al., 2021; Fan et al., 2024; Wang et al., 2022). The RMS is considered a reliable indicator of the variability indices of the COP movements and is defined as the square root of the mean of the squares of a sample (Hao et al., 2021; Paillard and Noé, 2015). Generally, postural stability deteriorated with higher linear indicator values. MSE can be used to identify COP fluctuations at multiple timescales; the higher the MSE value, the greater the COP complexity and the higher the adaptation of the postural control system (Busa and van Emmerik, 2016; Manor et al., 2010). In accordance with previous studies (Gow et al., 2015; Wang G. et al., 2024), we calculated the MSE of COP complexity in both the medial-lateral (ML) and anterior–posterior (AP) directions, used a scale factor of 8, and set the parameters *m* to 2 and *r* to 0.15.

For discrete wavelet transform analysis, the phases of interest were defined as adaptation (20–56 s) and reintegration (56–92 s), with each phase lasting 36 s (Wodarski et al., 2020). The COP data were split into four frequency bands using a 12-level Symlet-8 wavelet: (1) ultralow frequency band (< 0.10 Hz), (2) very-low-frequency band (0.10–0.39 Hz), (3) low-frequency band (0.39–1.56 Hz), and (4) moderate-frequency band (1.56–6.25 Hz). These frequency bands were calculated and represented as a percentage of total energy (Lin et al., 2019), corresponding to postural movements associated with the visual system (ultralow), vestibular system (very-low), cerebellar system (low), and muscular proprioception (moderate) (Cui et al., 2024; Lafleur and Lajoie, 2023; St-Amant et al., 2020).

### 2.4 Statistical analysis

Based on results from Craig et al. (2017) and Craig and Doumas (2019), a statistical power analysis indicated that a sample size of 10 would be sufficient to replicate the postural perturbation responses observed when a previously sway-referenced platform is stabilized (*α* = 0.05, power = 0.08). Thus, 24 participants in each group were considered adequate for the current study. Statistical analyses were performed using SPSS software (version 24.0; IBM Corp., Armonk, NY, United States). Normality tests (Shapiro–Wilk test) were performed. The independent *t*-test (for normally distributed data) and the Mann–Whitney U test (for non-normally distributed data) were used to assess differences in baseline participant characteristics. Values are presented as mean ± standard deviation or % (n).

First, to investigate postural stability and adaptability, a two-way repeated-measures analysis of variance (ANOVA) was used to identify the main and interaction effects of group and sensory perturbation on COP sway (velocity and RMS) and COP complexity (MSE_ML and MSE_AP). Multiple *t*-tests with Bonferroni corrections were used for follow-up comparisons (Craig and Doumas, 2019). We set the significance level at 0.05 with Bonferroni correction (*p* < 0.05/number of comparisons) for family-wise error to minimize the likelihood of Type I statistical errors (Magnard et al., 2020; Moccia et al., 2021; Nocera et al., 2020). Second, to investigate the speed of sensory reweighting, the phases of interest were defined as follows: baseline (BS window; 8–20 s), adaptation (A1, A2, and A3 windows; 20–32 s, 32–44 s, and 44–56 s, respectively), and reintegration (R1, R2, and R3 windows; 56–68 s, 68–80 s, and 80–92 s, respectively) (Doumas and Krampe, 2010; Wang et al., 2022). Two separate two-way repeated-measures ANOVA models were used to determine the main and interaction effects of group and time window on COP parameters during the adaptation (BS, A1, A2, and A3) and reintegration (BS, R1, R2, and R3) phases under each sensory perturbation condition. Multiple *t*-tests with Bonferroni correction (*p* < 0.05/number of comparisons) were used for follow-up comparisons. Effect sizes were reported for the ANOVAs using partial eta-squared (*η_p_*^2^), with *η_p_*^2^ values of 0.01–0.059 representing a small effect, 0.06–0.139 representing a medium effect, and > 0.140 representing a large effect (Cohen, 1973; Moccia et al., 2021). Third, to investigate the flexibility of sensory reweighting, the independent-samples *t*-test (for normally distributed data) and Mann–Whitney U test (for non-normally distributed data) were used to determine differences in the contribution of wavelet frequency bands during adaptation (20–56 s) and reintegration (56–92 s) between the groups. A significance level (*α*) of 0.05 was chosen for statistical comparisons after Bonferroni adjustment of the *p* values. Finally, the chi-squared test was used to evaluate the group differences in self-motion perception.

## 3 Results

### 3.1 Participant characteristics

One participant in the control group did not complete all tasks owing to vertigo. Finally, 47 participants completed the study, and their characteristics are presented in Supplementary Table S1. No significant differences in age, sex, height, weight, body mass index, MoCA, or BBS scores were observed between the groups. On average, the TC group (*n* = 24) reported 10.73 ± 4.84 years of TC experience (range 6–20 years) and the control group (*n* = 23) reported 11.30 ± 4.72 years of general aerobic exercise experience (range 6–20 years).

### 3.2 Postural stability and adaptability

**Figure 2:**
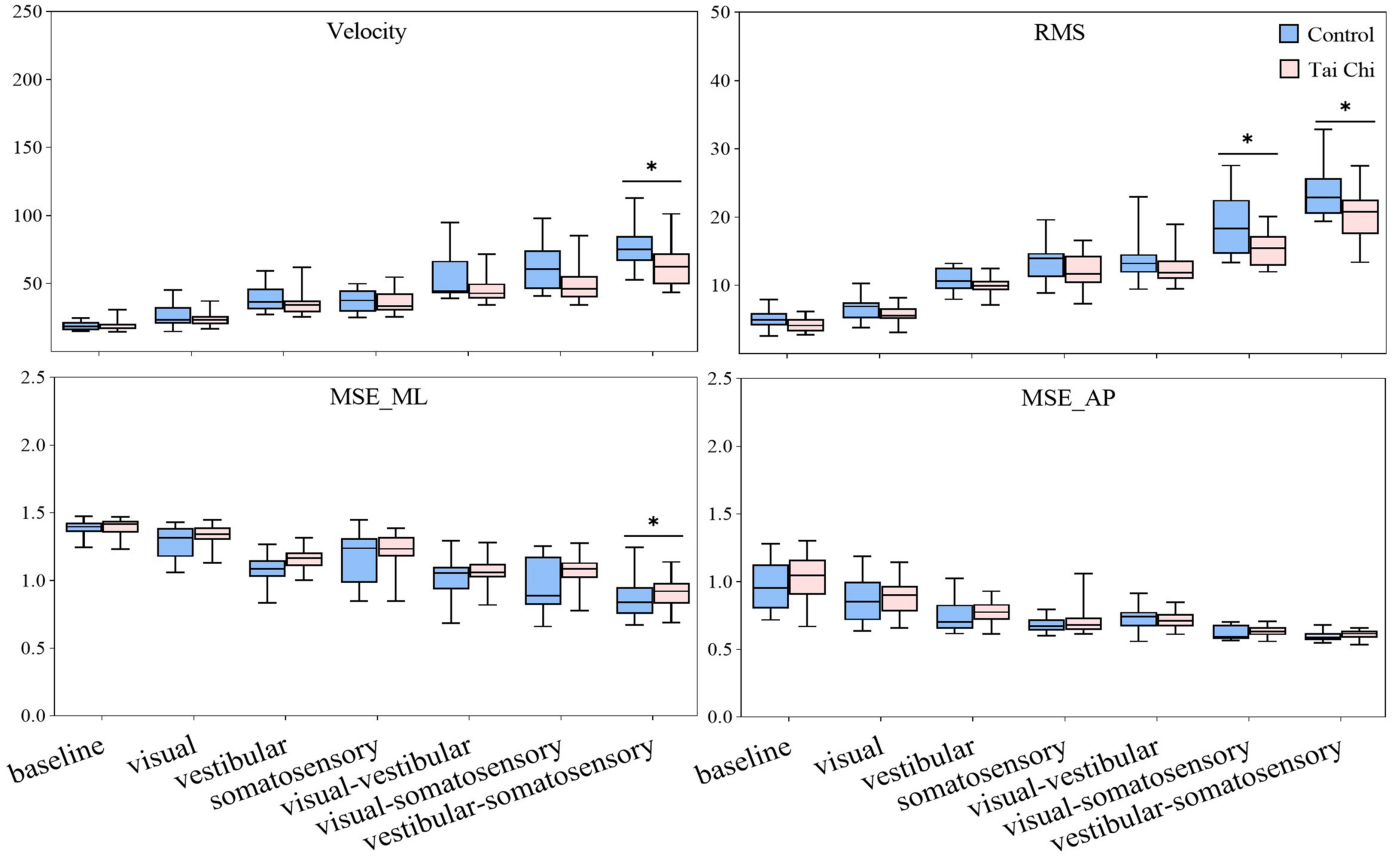
Comparative analysis of center-of-pressure sway (velocities and RMS) and complexity (MSE_ML and MSE_AP) during baseline and six sensory perturbations between the control (*n* = 23) and Tai Chi (*n* = 24) groups. The middle line in the box plots represents the median, while the top and bottom edges of the box correspond to the upper (75th percentile) and lower (25th percentile) quartiles, respectively. The upper and lower error bars indicate the maximum and minimum values of the data beyond the quartiles. *Significant difference between the groups, indicated by two-tailed independent-samples *t*-tests with an *α* level corrected for multiple comparisons (*p* < 0.00714). RMS, root mean square; MSE_ML, multiscale entropy medial-lateral; MSE_AP, multiscale entropy anterior–posterior.

Significant interaction effects of group with sensory perturbation for velocity and RMS were observed (*F* = 3.195–5.977, *p* < 0.05, *η_p_*^2^ = 0.066–0.117) suggesting that the TC group may show smaller velocity and RMS increase than the control group (Figure 2). Paired-samples *t*-tests, with an *α* level corrected for multiple comparisons to 0.00417 (i.e., 0.05/12), showed that both groups exhibited significantly higher velocity and RMS increases under all sensory perturbations than the baseline non-perturbation, especially multisensory perturbations (all *p* < 0.001). Two-tailed independent-samples *t*-tests, with an *α* level corrected for multiple comparisons to 0.00714 (i.e., 0.05/7), showed that the TC group exhibited smaller velocity under vestibular-somatosensory perturbation (*p* = 0.002) and smaller RMS under visual-somatosensory (*p* = 0.002) and vestibular-somatosensory perturbations (*p* = 0.003) than the control group, suggesting that TC practitioners exhibited better postural stability as the difficulty of standing tasks increased.

There was no interaction effect of group with sensory perturbation on MSE_ML and MSE_AP. Significant main effects of sensory perturbation (*F* = 201.561, *p* < 0.001, *η_p_*^2^ = 0.817) and group (*F* = 4.157, *p* = 0.047, *η_p_*^2^ = 0.085) were observed for MSE_ML. Significant main effects of sensory perturbation were also observed for MSE_AP (*F* = 153.440, *p* < 0.001, *η_p_*^2^ = 0.773). Paired-samples *t*-tests, with an *α* level corrected for multiple comparisons to 0.00417 (i.e., 0.05/12), showed that both groups exhibited significantly lower MSE_ML and MSE_AP under all sensory perturbations compared to baseline non-perturbation (all *p* ≤ 0.001), except visual perturbation. Under visual perturbation, the MSE_ML of the TC group did not show a significant difference compared to baseline non-perturbation, while the MSE_ML of the control group was significantly lower than that of the baseline non-perturbation (*p* < 0.001). This indicates that, compared to TC practitioners, postural adaptability among older adults in the control group is more significantly affected by visual perturbation. Two-tailed independent-samples *t*-tests, with an α level corrected for multiple comparisons to 0.00714 (i.e., 0.05/7), showed that the TC group exhibited higher MSE_ML under vestibular-somatosensory perturbation (*p* = 0.007) compared to the control group, suggesting that TC practitioners demonstrated better postural adaptability under challenging sensory conditions.

### 3.3 Amplitudes and durations of postural aftereffects during adaptation and reintegration

#### 3.3.1 Adaptation

**Figure 3:**
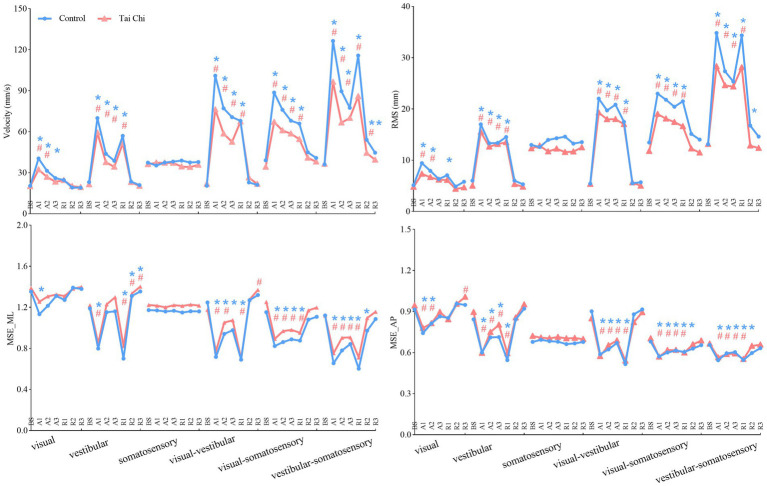
Comparative analysis of the time windows required for the center-of-pressure sway (velocities and RMS) and MSE (MSE_ML and MSE_AP) during the adaptation (A1–A3) and reintegration (R1–R3) phases under six sensory perturbations to return to BS levels for each group. For statistical analyses, the phases of interest were defined as: baseline (BS window, 8–20 s), adaptation (A1, A2, and A3 windows; 20–32 s, 32–44 s, and 44–56 s, respectively), and reintegration (R1, R2, and R3 windows; 56–68 s, 68–80 s, and 80–92 s, respectively). *^#^Significant difference between the time windows during adaptation (A1–A3)/reintegration (R1–R3) and the BS time window between the groups, indicated by paired-samples *t*-tests with an α level corrected for multiple comparisons (*p* < 0.0083). Unrecovered to BS levels in the control group, *p < 0.0083; Unrecovered to BS levels in the Tai Chi group, ^#^*p* < 0.0083. RMS, root mean square; MSE_ML, multiscale entropy medial-lateral; MSE_AP, multiscale entropy anterior–posterior.

**Figure 4:**
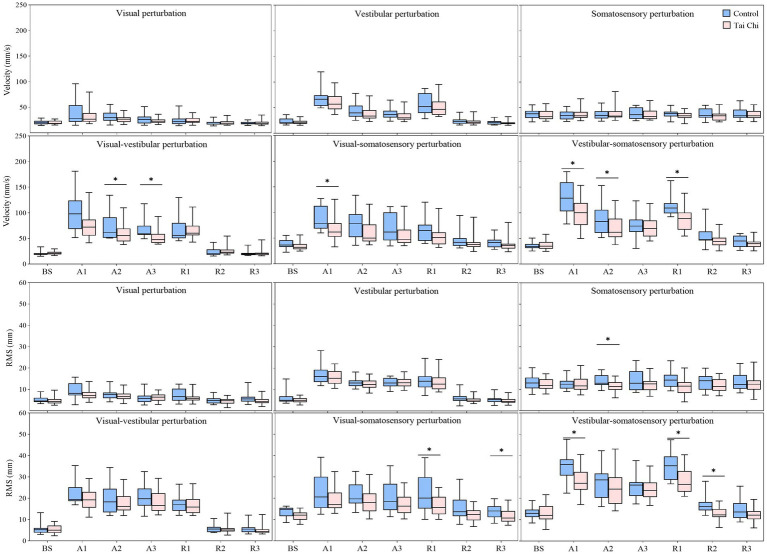
Comparative analysis of the center-of-pressure sway (velocities and RMS) during the adaptation (A1–A3) and reintegration (R1–R3) phases under six sensory perturbations between the control (*n* = 23) and Tai Chi (*n* = 24) groups. The middle line in the box plots represents the median, while the top and bottom edges of the box correspond to the upper (75th percentile) and lower (25th percentile) quartiles, respectively. The upper and lower error bars indicate the maximum and minimum values of the data beyond the quartiles. *Significant difference between the groups, indicated by two-tailed independent-samples *t*-tests with an α level corrected for multiple comparisons (*p* < 0.0125). RMS, root mean square.

Significant interaction effects of group with time window on COP parameters were observed during sensory perturbations (Supplementary Table S2). Paired-samples *t*-tests, with an *α* level corrected for multiple comparisons to 0.0083 (i.e., 0.05/6), showed that both groups exhibited significantly higher velocity and RMS under the A1 time window than those under the BS time window under all sensory perturbations (all *p* ≤ 0.008) except somatosensory perturbation (Figure 3, A1–A3 time windows). Despite the adverse impact of a sudden introduction of visual perturbation on velocity, the TC group exhibited shorter time windows to return to BS levels as the duration progressed than the control group (A2 vs. A3). Two-tailed independent-samples *t*-tests, with an α level corrected for multiple comparisons to 0.0125 (i.e., 0.05/4), showed that the TC group exhibited smaller velocity during the A2–A3 time windows under visual-vestibular perturbation, during the A1 time window under visual-somatosensory perturbation, and during the A1–A2 time windows under vestibular-somatosensory perturbation, compared to those of the control group (all *p* ≤ 0.0120). Similarly, the TC group exhibited smaller RMS during the A2 time window under somatosensory perturbation and during the A1 time window under vestibular-somatosensory perturbation than the control group (all *p* ≤ 0.009) (Figure 4, A1–A3 time windows).

**Figure 5:**
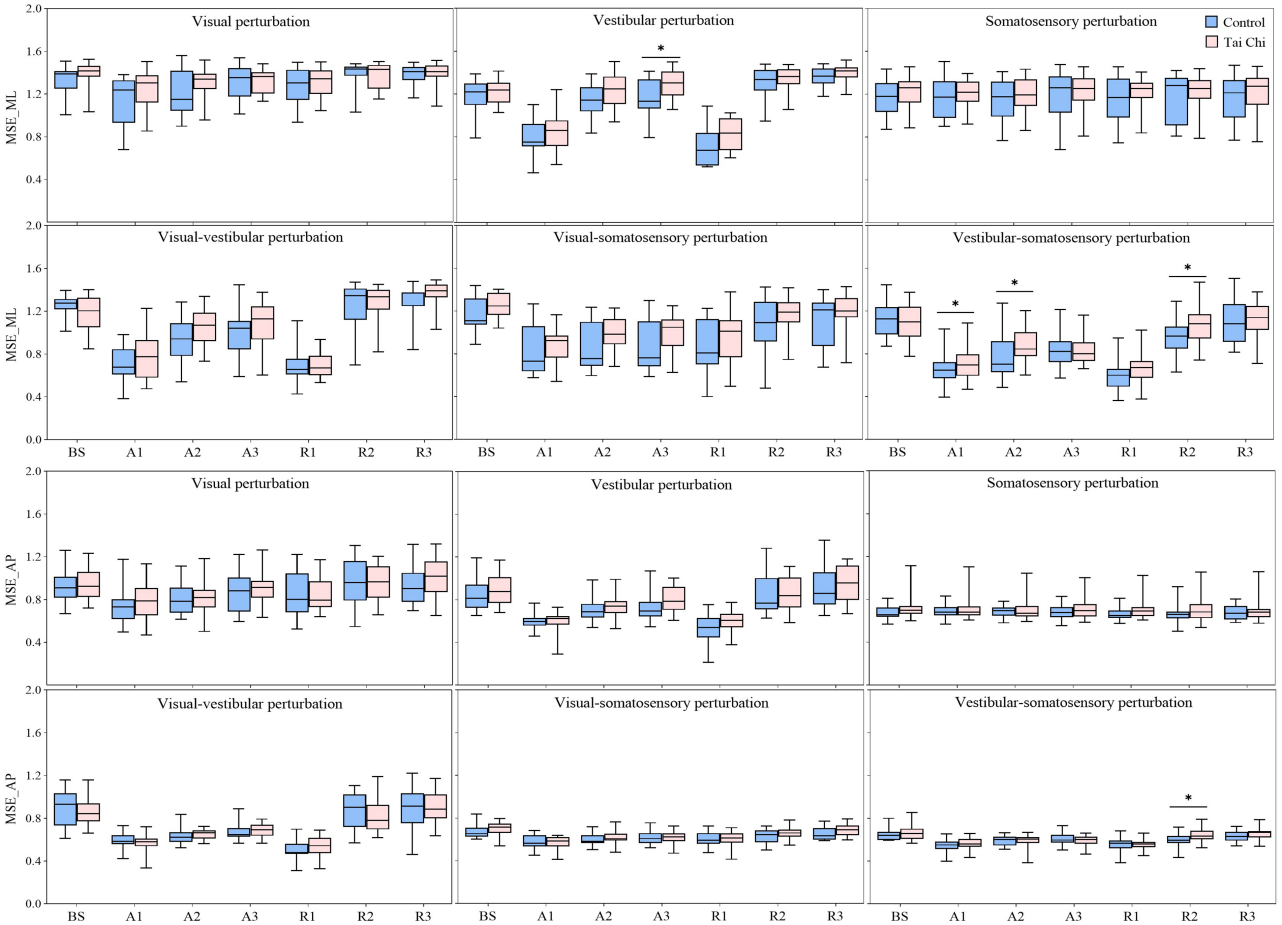
Comparative analysis of the center-of-pressure complexity (MSE_ML and MSE_AP) during the adaptation (A1–A3) and reintegration (R1–R3) phases under six sensory perturbations between the control (*n* = 23) and Tai Chi (*n* = 24) groups. The middle line in the box plots represents the median, while the top and bottom edges of the box correspond to the upper (75th percentile) and lower (25th percentile) quartiles, respectively. The upper and lower error bars indicate the maximum and minimum values of the data beyond the quartiles. *Significant difference between the groups, indicated by two-tailed independent-samples *t*-tests with an α level corrected for multiple comparisons (*p* < 0.0125). MSE_ML, multiscale entropy medial-lateral; MSE_AP, multiscale entropy anterior–posterior.

Additionally, the paired-samples *t*-tests, with an *α* level corrected for multiple comparisons to 0.0083 (i.e., 0.05/6), showed that both groups exhibited significantly lower MSE_ML and MSE_AP under the A1 time window compared to that of the BS time window under all sensory perturbations (all *p* ≤ 0.008) except visual and somatosensory perturbations (Figure 3, A1–A3 time windows). Despite the adverse impact of a sudden introduction of visual-vestibular perturbation on MSE_ML, the TC group exhibited smaller time windows to return to BS levels as the duration progressed than the control group (A3 vs. unrecovered). Under visual perturbation, the MSE_ML during the A1 time window of the TC group did not show a significant difference compared to that of the BS time window, while the MSE_ML during the A1 time window of the control group was significantly lower than that of the BS time window (*p* < 0.001). Two-tailed independent-samples *t*-tests, with an α level corrected for multiple comparisons to 0.0125 (i.e., 0.05/4), showed that the TC group exhibited higher MSE_ML during the A3 time window under vestibular perturbation and during the A1–A2 time windows under vestibular-somatosensory perturbation than the control group (all *p* ≤ 0.0123) (Figure 5, A1–A3 time windows). These results indicate that TC practitioners exhibited smaller amplitudes and shorter durations of postural aftereffects over time, which may reflect relatively quicker sensory reweighting during adaptation.

#### 3.3.2 Reintegration

There were significant interaction effects of group with time window on COP parameters during vestibular-somatosensory perturbation, as well as significant main effects of sensory perturbation and/or group on COP parameters under other sensory perturbations (Supplementary Table S3). Paired-samples *t*-tests, with an α level corrected for multiple comparisons to 0.0083 (i.e., 0.05/6), showed that both groups exhibited significantly higher velocity and RMS, with lower MSE_ML and MSE_AP under the R1 time window than those under the BS time window under most sensory perturbations (Figure 3, R1–R3 time windows). Despite the sudden cessation of sensory perturbations having a significant adverse impact on postural stability and adaptability in both groups, the time windows for recovery to BS levels differed between the groups. Specifically, this was observed in RMS (R1 vs. R2) under visual perturbation; in MSE_AP (R2 vs. R3) under visual-somatosensory perturbation; and in velocity (R3 vs. unrecovered), RMS (R2 vs. R3), MSE_ML (R2 vs. R3), and MSE_AP (R2 vs. R3) under vestibular-somatosensory perturbation, with the TC group showing earlier recovery. The TC group also exhibited significantly higher MSE_ML in the R3 time window after the cessation of visual-vestibular perturbation and higher MSE_AP in the R3 time window after the cessation of visual perturbation compared to BS levels, while the control group only returned to BS levels.

Two-tailed independent-samples *t*-tests, with an α level corrected for multiple comparisons to 0.0125 (i.e., 0.05/4), showed that the TC group exhibited smaller velocity during the R1 time window under vestibular-somatosensory perturbation, smaller RMS during the R1 and R3 time windows under visual-somatosensory perturbation, and during the R1–R2 time windows under vestibular-somatosensory perturbation, compared to those of the control group (all *p* ≤ 0.01) (Figure 4, R1–R3 time windows). Similarly, the TC group exhibited higher MSE_ML during the R2 time window under vestibular-somatosensory perturbation and higher MSE_AP during the R2 time window under vestibular-somatosensory perturbation, than the control group (all *p* ≤ 0.0120) (Figure 5, R1–R3 time windows). Both groups experienced a substantial aftereffect of increased COP sway and decreased COP complexity; however, these aftereffects were shorter in duration and smaller in magnitude for the TC group, suggesting that TC practitioners have a relatively faster sensory reweighting process during reintegration.

### 3.4 Weighting of the sensory systems

#### 3.4.1 Adaptation

**Figure 6:**
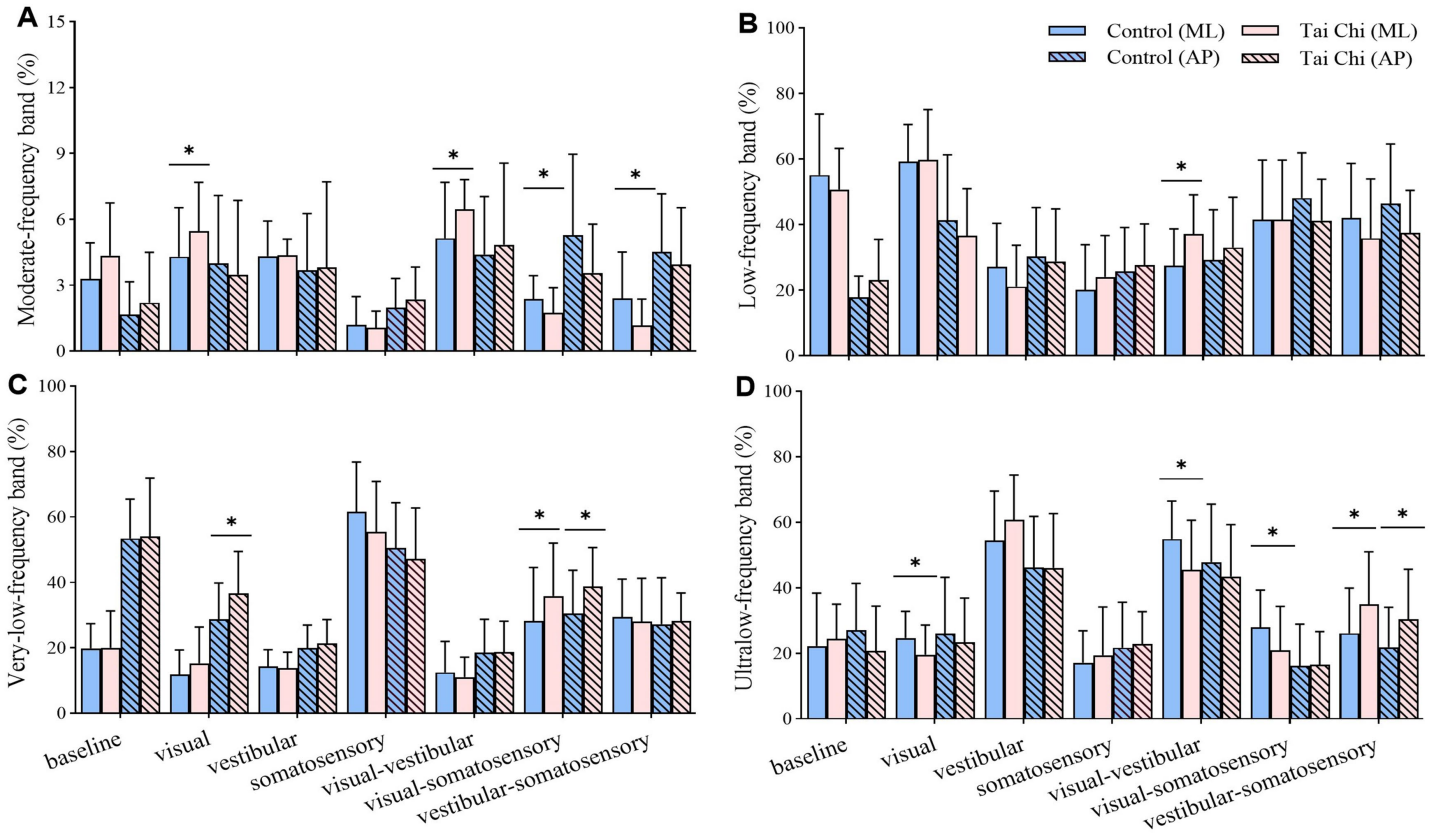
Comparative analysis of the percentage of energy content in each frequency band during adaptation (20–56 s) under different standing tasks between the control (*n* = 23) and Tai Chi (*n* = 24) groups. **(A)** Moderate-frequency band (1.56–6.25 Hz, proprioception inputs); **(B)** Low-frequency band (0.39–1.56 Hz, cerebellar system); **(C)** Very-low-frequency band (0.10–0.39 Hz, vestibular system); **(D)** Ultralow-frequency band (< 0.10 Hz, visual system). The independent *t*-test (for normally distributed data) and the Mann–Whitney U test (for non-normally distributed data) were used to assess group differences. A significance level (α) of 0.05 was chosen for statistical comparisons after Bonferroni adjustment of the *p* values, *p < 0.05; ML, medial-lateral; AP, anterior–posterior.

Compared with the control group, the TC group exhibited (1) higher energy content of moderate_ML-frequency (proprioception system) and very-low_AP-frequency band (vestibular system) but lower ultralow_ML-frequency band (visual system) under visual perturbation; (2) higher energy content of moderate_ML-frequency (proprioception system) and low_ML-frequency band (cerebellar system) but lower ultralow_ML-frequency band (visual system) under visual-vestibular perturbation; (3) higher energy content of very-low_ML-and very-low_AP-frequency band (vestibular system) but lower moderate_ML-frequency (proprioception system) and ultralow_ML-frequency band (visual system) under visual-somatosensory perturbation; and (4) higher energy content of ultralow_ML-and ultralow_AP-frequency band (visual system) but lower moderate_ML-frequency band (proprioception system) under vestibular-somatosensory perturbation (Figure 6).

#### 3.4.2 Reintegration

**Figure 7:**
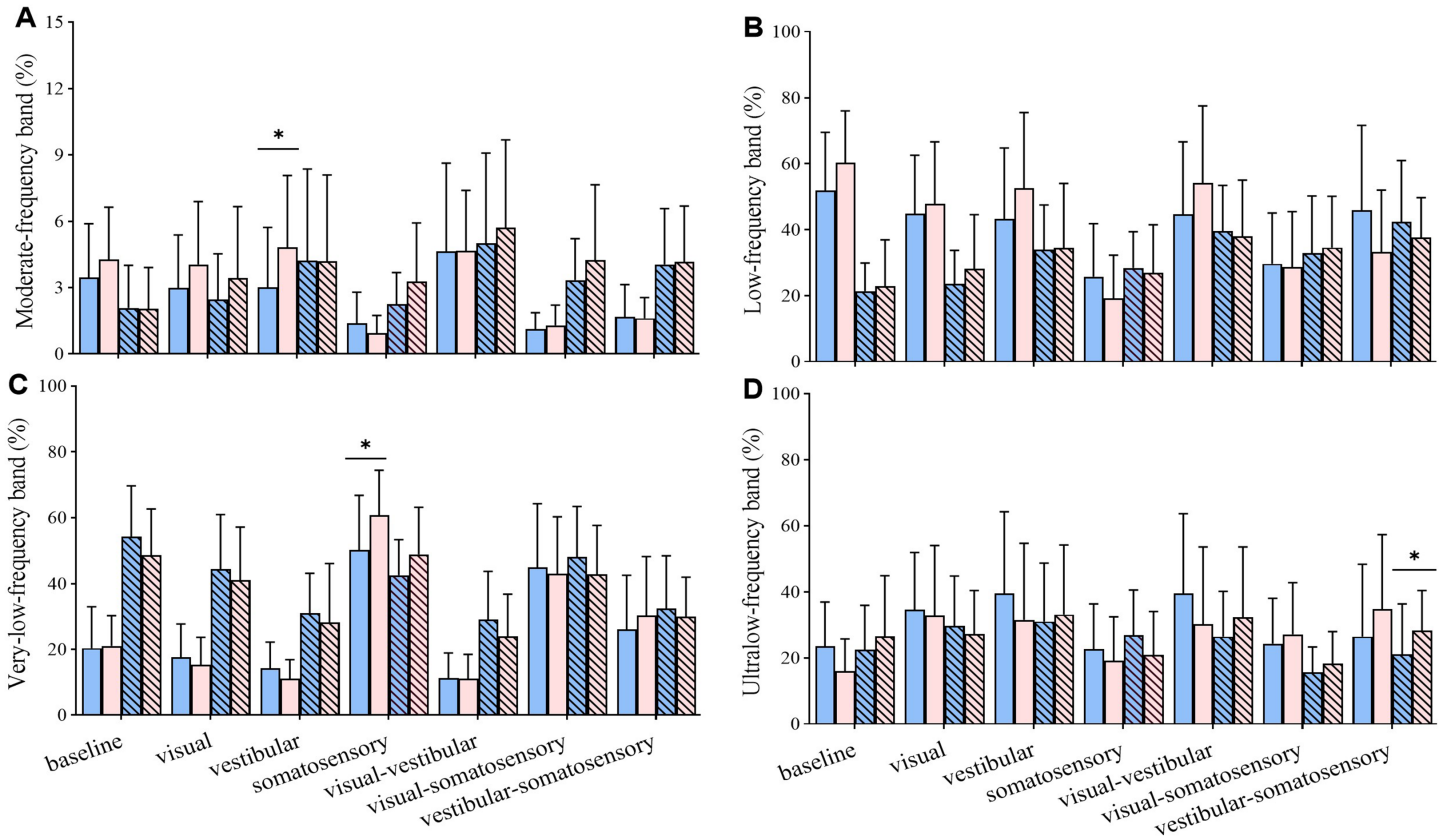
Comparative analysis of the percentage of energy content in each frequency band during reintegration (56–92 s) under different standing tasks between the control (*n* = 23) and Tai Chi (*n* = 24) groups. **(A)** Moderate-frequency band (1.56–6.25 Hz, proprioception inputs); **(B)** Low-frequency band (0.39–1.56 Hz, cerebellar system); **(C)** Very-low-frequency band (0.10–0.39 Hz, vestibular system); **(D)** Ultralow-frequency band (< 0.10 Hz, visual system). The independent *t*-test (for normally distributed data) and the Mann–Whitney U test (for non-normally distributed data) were used to assess group differences. A significance level (α) of 0.05 was chosen for statistical comparisons after Bonferroni adjustment of the *p* values, *p < 0.05; ML, medial-lateral; AP, anterior–posterior.

Compared with the control group, the TC group exhibited (1) higher energy content of moderate_ML-frequency band (proprioception system) under vestibular perturbation; (2) higher energy content of very-low_ML-frequency band (vestibular system) under somatosensory perturbation; and (3) higher energy content of ultralow_AP-frequency band (visual system) under vestibular-somatosensory perturbation (Figure 7).

### 3.5 Self-motion perception

Compared with the baseline, all sensory perturbations, except somatosensory perturbation, had detrimental effects on self-motion perception accuracy (*p* < 0.05). The TC group exhibited significantly higher accuracy during visual (*p* = 0.049), vestibular (*p* = 0.041), and visual-vestibular (*p* = 0.036) perturbations than did the control group (Supplementary Table S4).

## 4 Discussion

This cross-sectional study explored the differences in sensory reweighting and self-motion perception for postural control under single-sensory and multisensory perturbations between older TC practitioners and healthy active older controls. Consistent with our hypothesis, TC practitioners, compared to non-practitioners, exhibited: (1) superior postural stability and adaptability; (2) a relatively faster sensory reweighting process, indicated by smaller amplitudes and shorter durations of postural aftereffects during adaptation and reintegration; (3) higher flexibility in sensory reweighting, indicated by increased reliance on unperturbed sensory systems and decreased reliance on perturbed; and (4) enhanced self-motion perception, reflected by higher self-motion perception accuracy during visual, vestibular, and visual-vestibular perturbations. These findings partly shed light on the underlying mechanisms of better postural stability and adaptability in older TC practitioners.

We analyzed the participants’ COP sway (velocity and RMS) and COP complexity (MSE_ML and MSE_AP) under baseline non-perturbation and six sensory perturbations to compare the differences in postural stability and adaptability between the groups. We found no significant differences in all COP parameters between the groups under baseline non-perturbation. Compared to the baseline condition, single-sensory and multisensory perturbations had significant adverse effects on postural control, indicating that sensory perturbations, especially multisensory perturbations, challenged the postural control systems of both groups. Although a decreased balance performance was observed in both groups during challenging sensory conditions, TC practitioners exhibited significantly smaller COP sway and higher COP complexity than non-practitioners as the difficulty of standing tasks increased. This suggests that TC practitioners have better ability to resist challenging sensory perturbations to maintain superior postural stability and adaptability. In contrast, no significant group difference was observed in the COP parameters under simple, less challenging conditions, such as somatosensory perturbation. This implies that relatively less challenging perturbation paradigms cannot effectively differentiate postural control between older TC practitioners and non-practitioners during prolonged standing tasks. Increasing the difficulty of prolonged tasks may be necessary for such differentiation. Previous studies primarily utilized long-duration static or constant perturbations, which allowed participants to reach steady-state conditions owing to their adaptive capacity (Doumas and Krampe, 2010). This may not sensitively detect the effects of TC on postural control, which could explain why some studies showed reductions in COP sway among TC practitioners when standing (Chen et al., 2012; Guan and Koceja, 2011; Tsang et al., 2004), whereas others reported no significant changes in this parameter (Chen et al., 2011; Cui et al., 2024; Wayne et al., 2014).

Sensory reweighting, a mechanism highly sensitive to age-related decline, is often inefficient in older adults (Craig and Doumas, 2019; Doumas and Krampe, 2010). Older adults have a larger integration time window compared to younger adults, indicating slower sensory reweighting (Craig and Doumas, 2019; Doumas and Krampe, 2010; Zhang et al., 2020). To assess the differences in the speed of sensory reweighting between the two groups, we mapped out time courses of COP sway and COP complexity recovery using the time-window-of-integration model. We found that for significant interaction effects of group with time window on COP parameters during adaptation and reintegration, the time window required to return to the BS time window was smaller in TC practitioners than that in non-practitioners. For example, during adaptation, the TC group had a smaller time window for recovery to BS levels under visual-vestibular perturbation for MSE_ML. During reintegration, the TC group showed earlier recovery in velocity, RMS, MSE_ML, and MSE_AP under vestibular-somatosensory perturbation. The smaller amplitudes and shorter durations of postural aftereffects under sensory perturbation reflect quicker sensory reweighting (Craig and Doumas, 2019; Doumas and Krampe, 2010; Peterka and Loughlin, 2004). This finding suggests that long-term TC practitioners may have a relatively faster sensory reweighting process for postural control.

Sensory reweighting is a major contributor to limiting body sway amplitudes when balance is perturbed (Craig and Doumas, 2019). However, older adults have reduced flexibility in multisensory interaction (Yang et al., 2023). We used modern mathematical methods to examine the differences in the weighting of sensory systems for postural control under sensory perturbations between the groups. This method can simultaneously consider the time and frequency domains in the signal process (Lin et al., 2019). In discrete wavelet analysis, the energy content is decomposed into four distinct frequency bands, which represent the proprioceptive, cerebellar, vestibular, and visual systems (Lin et al., 2019). This quadripartite classification can provide a comprehensive understanding of the sensory system in various challenging tasks. Frequency analysis showed that TC practitioners exhibited significantly increased weighting on unperturbed sensory systems and decreased weighting on perturbed sensory inputs than non-practitioners, particularly during the adaptation phase. This suggests that older adults with long-term TC experience may exhibit flexible sensory reweighting for postural control, reducing reliance on unreliable sensory information while increasing reliance on other reliable inputs. This partially supports the findings of a recent study (Cui et al., 2024), which reported that long-term TC practice improved sensory reweighting by increasing reliance on the proprioception system and decreasing reliance on the vestibular system during standing balance with eyes closed. Our results also suggest the possibility of multiple compensatory sensory weighting patterns in postural control among long-term TC practitioners.

Self-motion perception is the individual’s coherent perceptual experience of their own body based on information from the different senses (Chancel et al., 2022; Stein and Stanford, 2008). Therefore, self-motion perception can reflect multisensory integration ability (Stein and Stanford, 2008; Wang et al., 2023). Previous studies have suggested that the human brain gives different weights to the information from different sensory modalities and, in some cases, information in a certain sensory modality is preferentially processed and occupies a dominant position, leading to multisensory illusion effects, which may further increase postural instability (Chen and Zhou, 2013; Craig and Doumas, 2019; Spence, 2011). For instance, older adults generally exhibit higher visual weighting and a slower decline in visual weight (Jeka et al., 2010). This visual dependence tends to cause older adults to experience errors in perceptual judgments more frequently compared to younger adults, and it has been associated with balance decline and higher risk of falls (Chancel et al., 2018; Costello and Bloesch, 2017; Gabriel et al., 2022; Poulain and Giraudet, 2008). In this study, TC practitioners exhibited significantly higher self-motion perception accuracy than did non-practitioners under visual, vestibular, and visual-vestibular perturbations, suggesting that they were less susceptible to irrelevant visual and/or vestibular information in a reliable somatosensory environment. This may reflect reduced visual dependence in older adults with long-term TC experience. This change is also evident in wavelet band analysis, where TC practitioners showed lower visual input weights during visual perturbation compared to those of non-practitioners. However, both groups were equally affected by irrelevant visual or vestibular information in unreliable somatosensory environments. This suggests that proprioceptive information plays a crucial role in maintaining accurate self-motion perception among older TC practitioners. Overall, long-term TC practitioners have superior postural stability and adaptability, especially under more challenging sensory perturbations. The underlying causes may include a relatively faster and flexible sensory reweighting process and higher perception ability. These improvements support that TC may be a balance exercise that helps improve multisensory integration mechanisms (Wang et al., 2023).

This study has several strengths. First, we used traditional linear and novel nonlinear methods that could provide complementary information for evaluating postural control and potential mechanisms in older TC practitioners. Second, our results provide evidence of changes in the speed and nature of sensory reweighting in older TC practitioners. By setting up multiple challenging changing sensory environments and using the time-window-of-integration model, we created additional demands on dynamic sensory reweighting and postural control. This approach offers a more comprehensive understanding of the effects of TC on sensory reweighting and balance control. Third, we assessed self-motion perception for postural control using subjective reports, which could reflect changes in the efficacy of multisensory integration. Our study also has some limitations. As an exploratory study, the cross-sectional design used does not allow for definitive conclusions regarding the cause-and-effect relationship between long-term TC practice and improvements in sensory reweighting and self-motion perception for postural control in older adults. Future interventional trials are needed to verify our findings. We also acknowledge the statistics that family-wise error rates and potential false positive findings may increase; thus, these results should be interpreted with caution. Another limitation of this study is that the age range of the older adults is relatively broad. Thus, some confounding effect of age may not able rule out. Although a soft surface can disrupt somatosensory inputs, as shown in previous studies (Li et al., 2019; Bai et al., 2023), it may not present a sudden challenge to the somatosensory system. Future studies should address this limitation by incorporating transient somatosensory perturbations (Wang D. et al., 2024). Furthermore, dynamic changes in sensory reweighting were inferred from COP measures. Future studies should analyze the neural mechanisms related to multisensory integration behind the improvement of postural control by TC by applying neuroimaging techniques, such as functional near-infrared spectroscopy and electroencephalography (Li et al., 2024). Despite these limitations, our study still provides valuable preliminary evidence suggesting that TC practitioners exhibit better postural control, and the underlying mechanisms may include improved sensory weighting and perceptual abilities.

## 5 Conclusion

Long-term TC practitioners exhibited superior postural stability and adaptability under challenging sensory perturbations, along with smaller amplitudes and shorter durations of postural aftereffects over time during adaptation and reintegration. These improvements may be attributed to a relatively rapid and flexible sensory reweighting and improved self-motion perception for postural control. Given that falls have become a critical global challenge, addressing the effects of TC as an exercise intervention to enhance sensory reweighting and self-motion perception for postural control in older adults may be an exciting research avenue with the potential for public health impact.

## Data Availability

The original contributions presented in the study are included in the article/Supplementary material, further inquiries can be directed to the corresponding author.
