# Supplementary material for: Sensory reweighting and self-motion perception for postural control under single-sensory and multisensory perturbations in older Tai Chi practitioners
Source: Front Hum Neurosci. 2024 Nov 1;18:1482752. doi: 10.3389/fnhum.2024.1482752 (PMC11565703; doi:10.3389/fnhum.2024.1482752)
Supplement: Supplementary file 1 [file Table_1.docx]

Supplementary Material

**Supplementary Table 1.** Participant characteristics

| Measure | Control (*n* = 23) | Tai Chi (*n* = 24) | p |
| --- | --- | --- | --- |
| Age (years) | 67.26 ± 5.71 | 67.79 ± 5.16 | 0.740 |
| Sex (male) | 47.83% (11) | 29.17% (7) | 0.238 |
| Height (cm) | 162.02 ± 4.93 | 160.83 ± 5.05 | 0.380 |
| Weight (kg) | 62.40 ± 6.22 | 59.06 ± 7.54 | 0.081 |
| BMI (kg/m^2^) | 23.76 ± 2.02 | 22.84 ± 2.67 | 0.191 |
| MoCA | 26.08 ± 1.32 | 26.30 ± 1.37 | 0.583 |
| BBS | 55.83 ± 0.39 | 55.92 ± 0.28 | 0.363 |

*Note:* Mean ± standard deviation or % (n). BMI, body mass index; MoCA, Montreal Cognitive Assessment; BBS, Berg Balance Scale.

**Supplementary Table 2.** Main and interaction effects of group and time window on COP sway and COP complexity during adaptation

| Variables | **Single-sensory perturbations (*F*, *p*, *η*^2^)** | | |  | **Multisensory perturbations (*F*, *p*, *η*^2^)** | | |
| --- | --- | --- | --- | --- | --- | --- | --- |
|  | Group | time window | Group*time window |  | Group | time window | Group*time window |
| Visual |  |  |  |  | Visual-vestibular |  |  |
| Velocity | *F*=2.345, *p*=0.133, *η*^2^=0.050 | *F*=32.339, ***p*<0.001**, *η*^2^=0.418 | *F*=1.828, *p*=0.180, *η*^2^=0.039 |  | *F*=7.433, ***p*=0.009**, *η*^2^=0.142 | *F*=143.751, ***p*<0.001**, *η*^2^=0.762 | *F*=5.714, ***p*=0.006**, *η*^2^=0.113 |
| RMS | *F*=3.503, *p*=0.068, *η*^2^=0.072 | *F*=29.369, ***p*<0.001**, *η*^2^=0.395 | *F*=2.736, ***p*=0.046**, *η*^2^=0.057 |  | *F*=2.866, *p*=0.097, *η*^2^=0.060 | *F*=204.127, ***p*<0.001**, *η*^2^=0.819 | *F*=1.611, *p*=0.198, *η*^2^=0.035 |
| MSE_ML | *F*=3.608, *p*=0.064, *η*^2^=0.074 | *F*=17.214, ***p*<0.001**, *η*^2^=0.277 | *F*=1.890, *p*=0.145, *η*^2^=0.040 |  | *F*=1.407, *p*=0.242, *η*^2^=0.030 | *F*=101.733, ***p*<0.001**, *η*^2^=0.693 | *F*=4.688, ***p*=0.011**, *η*^2^=0.094 |
| MSE_AP | *F*=0.379, *p*=0.541, *η*^2^=0.008 | *F*=35.095, ***p*<0.001**, *η*^2^=0.438 | *F*=0.416, *p*=0.696, *η*^2^=0.009 |  | *F*=0.094, *p*=0.761, *η*^2^=0.002 | *F*=112.686, ***p*<0.001**, *η*^2^=0.715 | *F*=2.408, *p*=0.101, *η*^2^=0.051 |
| Vestibular |  |  |  |  | Visual-proprioceptive |  |  |
| Velocity | *F*=3.415, *p*=0.071, *η*^2^=0.071 | *F*=216.300, ***p*<0.001**, *η*^2^=0.828 | *F*=2.445, *p*=0.091, *η*^2^=0.052 |  | *F*=5.894, ***p*=0.019**, *η*^2^=0.116 | *F*=86.284, ***p*<0.001**, *η*^2^=0.657 | *F*=3.614, ***p*=0.015**, *η*^2^=0.074 |
| RMS | *F*=2.991, *p***=**0.091, *η*^2^=0.062 | *F*=163.600, ***p*<0.001**, *η*^2^=0.784 | *F*=0.558, *p*=0.590, *η*^2^=0.012 |  | *F*=7.703, ***p*=0.008**, *η*^2^=0.146 | *F*=36.822, ***p*<0.001**, *η*^2^=0.450 | *F*=0.679, *p*=0.567, *η*^2^=0.015 |
| MSE_ML | *F*=5.259, ***p*=0.027**, *η*^2^=0.105 | *F*=128.921, ***p*<0.001**, *η*^2^=0.741 | *F*=1.834, *p*=0.161, *η*^2^=0.039 |  | *F*=4.007, *p*=0.051, *η*^2^=0.082 | *F*=107.449, ***p*<0.001**, *η*^2^=0.705 | *F*=0.300, *p*=0.825, *η*^2^=0.007 |
| MSE_AP | *F*=2.423, *p*=0.127, *η*^2^=0.051 | *F*=81.012, ***p*<0.001**, *η*^2^=0.643 | *F*=2.448, *p*=0.083, *η*^2^=0.052 |  | *F*=0.656, *p*=0.422, *η*^2^=0.014 | *F*=47.849, ***p*<0.001**, *η*^2^=0.515 | *F*=0.599, *p*=0.617, *η*^2^=0.013 |
| Proprioceptive |  |  |  |  | Vestibular-proprioceptive |  |  |
| Velocity | *F*=0.002, *p*=0.964, *η*^2^=0.001 | *F*=0.630, *p*=0.597, *η*^2^=0.014 | *F*=0.937, *p*=0.425, *η*^2^=0.020 |  | *F*=7.168, ***p*=0.010**, *η*^2^=0.137 | *F*=188.547, ***p*<0.001**, *η*^2^=0.807 | *F*=9.626, ***p*<0.001**, *η*^2^=0.176 |
| RMS | *F*=2.359, *p*=0.132, *η*^2^=0.050 | *F*=0.740, *p*=0.530, *η*^2^=0.016 | *F*=3.127, ***p*=0.028**, *η*^2^=0.065 |  | *F*=4.258, ***p*=0.045**, *η*^2^=0.086 | *F*=142.258, ***p*<0.001**, *η*^2^=0.760 | *F*=4.960, ***p*=0.004**, *η*^2^=0.099 |
| MSE_ML | *F*=1.099, *p*=0.300, *η*^2^=0.024 | *F*=0.368, *p*=0.776, *η*^2^=0.008 | *F*=0.049, *p*=0.985, *η*^2^=0.001 |  | *F*=3.213, *p*=0.080, *η*^2^=0.067 | *F*=138.662, ***p*<0.001**, *η*^2^=0.755 | *F*=3.395, ***p*=0.028**, *η*^2^=0.070 |
| MSE_AP | *F*=1.450, *p*=0.235, *η*^2^=0.031 | *F*=0.329, *p*=0.805, *η*^2^=0.007 | *F*=1.329, *p*=0.267, *η*^2^=0.029 |  | *F*=0.074, *p*=0.787, *η*^2^=0.002 | *F*=34.527, ***p*<0.001**, *η*^2^=0.434 | *F*=0.900, *p*=0.431, *η*^2^=0.020 |

Note*:* COP, center-of-pressure; RMS, root mean square; MSE, multiscale entropy; ML, medial-lateral; AP, anterior-posterior. Significance levels are indicated in bold. Significant difference between the groups (*p* < 0.05).

**Supplementary Table 3.** Main and interaction effects of group and time window on COP sway and COP complexity during reintegration

| Variables | **Single-sensory perturbations (*F*, *p*, *η*^2^)** | | |  | **Multisensory perturbations (*F*, *p*, *η*^2^)** | | |
| --- | --- | --- | --- | --- | --- | --- | --- |
|  | Group | time window | Group*time window |  | Group | time window | Group*time window |
| Visual |  |  |  |  | Visual-vestibular |  |  |
| Velocity | *F*=0.018, *p*=0.893, *η*^2^=0.001 | *F*=23.575, ***p*<0.001**, *η*^2^=0.344 | *F*=0.387, *p*=0.667, *η*^2^=0.009 |  | *F*=0.198, *p*=0.659, *η*^2^=0.004 | *F*=256.292, ***p*<0.001**, *η*^2^=0.851 | *F*=0.384, *p*=0.564, *η*^2^=0.008 |
| RMS | *F*=3.025, *p*=0.089, *η*^2^=0.063 | *F*=13.143, ***p*<0.001**, *η*^2^=0.226 | *F*=0.581, *p*=0.594, *η*^2^=0.013 |  | *F*=0.257, *p*=0.615, *η*^2^=0.006 | *F*=302.452 ***p*<0.001**, *η*^2^=0.870 | *F*=0.315, *p*=0.660, *η*^2^=0.007 |
| MSE_ML | *F*=0.856, *p*=0.360, *η*^2^=0.019 | *F*=7.870, ***p*<0.001**, *η*^2^=0.149 | *F*=0.462, *p*=0.710, *η*^2^=0.010 |  | *F*=0.004, *p*=0.951, *η*^2^=0.001 | *F*=289.378, ***p*<0.001**, *η*^2^=0.865 | *F*=2.106, *p*=0.119, *η*^2^=0.045 |
| MSE_AP | *F*=0.149, *p*=0.701, *η*^2^=0.003 | *F*=15.127, ***p*<0.001**, *η*^2^=0.252 | *F*=1.190, *p*=0.316, *η*^2^=0.026 |  | *F*=0.754, *p*=0.390, *η*^2^=0.016 | *F*=116.161, ***p*<0.001**, *η*^2^=0.721 | *F*=1.383, *p*=0.256, *η*^2^=0.030 |
| Vestibular |  |  |  |  | Visual-somatosensory |  |  |
| Velocity | *F*=0.837, *p*=0.365, *η*^2^=0.018 | *F*=181.922, ***p*<0.001**, *η*^2^=0.802 | *F*=0.939, *p*=0.349, *η*^2^=0.020 |  | *F*=2.368, *p*=0.131, *η*^2^=0.050 | *F*=58.613, ***p*<0.001**, *η*^2^=0.566 | *F*=1.917, *p*=0.159, *η*^2^=0.041 |
| RMS | *F*=2.612, *p*=0.113, *η*^2^=0.055 | *F*=150.767, ***p*<0.001**, *η*^2^=0.770 | *F*=0.126, *p*=0.817, *η*^2^=0.003 |  | *F*=11.505, ***p*=0.001**, *η*^2^=0.204 | *F*=38.325, ***p*<0.001**, *η*^2^=0.460 | *F*=1.883, *p*=0.162, *η*^2^=0.040 |
| MSE_ML | *F*=4.867, ***p*=0.033**, *η*^2^=0.098 | *F*=274.961, ***p*<0.001**, *η*^2^=0.859 | *F*=1.798, *p*=0.161, *η*^2^=0.038 |  | *F*=3.135, *p*=0.083, *η*^2^=0.065 | *F*=61.730, ***p*<0.001**, *η*^2^=0.578 | *F*=0.087, *p*=0.930, *η*^2^=0.002 |
| MSE_AP | *F*=0.975, *p*=0.329, *η*^2^=0.021 | *F*=113.407, ***p*<0.001**, *η*^2^=0.716 | *F*=0.361, *p*=0.665, *η*^2^=0.008 |  | *F*=2.132, *p*=0.151, *η*^2^=0.045 | *F*=30.795, ***p*<0.001**, *η*^2^=0.406 | *F*=1.589, *p*=0.204, *η*^2^=0.034 |
| Somatosensory |  |  |  |  | Vestibular-somatosensory |  |  |
| Velocity | *F*=1.404, *p*=0.242, *η*^2^=0.030 | *F*=0.352, *p*=0.788, *η*^2^=0.008 | *F*=0.864, *p*=0.462, *η*^2^=0.019 |  | *F*=10.953, ***p*=0.002**, *η*^2^=0.196 | *F*=334.157, ***p*<0.001**, *η*^2^=0.881 | *F*=16.428, ***p*<0.001**, *η*^2^=0.267 |
| RMS | *F*=3.670, *p*=0.062, *η*^2^=0.075 | *F*=0.633, *p*=0.595, *η*^2^=0.014 | *F*=1.850, *p*=0.141, *η*^2^=0.039 |  | *F*=14.524, ***p*<0.001**, *η*^2^=0.244 | *F*=283.681, ***p*<0.001**, *η*^2^=0.863 | *F*=6.548, ***p*=0.002**, *η*^2^=0.127 |
| MSE_ML | *F*=1.349, *p*=0.252, *η*^2^=0.029 | *F*=0.301, *p*=0.787, *η*^2^=0.007 | *F*=0.082, *p*=0.951, *η*^2^=0.002 |  | *F*=3.858, *p*=0.056, *η*^2^=0.079 | *F*=211.714, ***p*<0.001**, *η*^2^=0.825 | *F*=3.368, ***p*=0.021**, *η*^2^=0.070 |
| MSE_AP | *F*=2.441, *p*=0.125, *η*^2^=0.051 | *F*=1.033, *p*=0.380, *η*^2^=0.022 | *F*=0.518, *p*=0.671, *η*^2^=0.011 |  | *F*=3.451, *p*=0.070, *η*^2^=0.071 | *F*=39.676, ***p*<0.001**, *η*^2^=0.469 | *F*=1.780, *p*=0.162, *η*^2^=0.038 |

Note*:* COP, center-of-pressure; RMS, root mean square; MSE, multiscale entropy; ML, medial-lateral; AP, anterior-posterior. Significance levels are indicated in bold. Significant difference between the groups (*p* < 0.05).

**Supplementary Table 4.** Spatial perception accuracy comparisons between the groups

| Perturbation | Control (*n* = 23) | Tai Chi (*n* = 24) | p |
| --- | --- | --- | --- |
| Baseline | 100% (23) | 100% (24) | 1.000 |
| Visual | 13.04% (3) | 41.67% (10) | **0.049** |
| Vestibular | 30.43% (7) | 62.5% (15) | **0.041** |
| Somatosensory | 100% (23) | 100% (24) | 1.000 |
| Visual-Vestibular | 8.70% (2) | 37.5% (9) | **0.036** |
| Visual-Somatosensory | 17.39% (4) | 37.5% (9) | 0.193 |
| Vestibular-Somatosensory | 34.78% (8) | 58.33% (14) | 0.147 |

*Note:* % (n). Significance levels are indicated in bold. Significant difference between the groups (*p* < 0.05).
